# Child exposure to domestic violence, substance dependence and suicide resilience in child laborers

**DOI:** 10.1186/s12889-023-15367-7

**Published:** 2023-03-10

**Authors:** Fateme Mohammadi, Khodayar Oshvandi, Farshid Shamsaei, Masoud Khodaveisi, Salman Khazaei, Seyedeh Zahra Masoumi

**Affiliations:** 1 Chronic Diseases (Home Care) Research Center and Autism Spectrum Disorders Research Center, Department of Nursing, Hamadan University of Medical Sciences, Hamadan, Iran; 2 Mother and Child Care Research Center, Department of Nursing, Hamadan University of Medical Sciences, Hamadan, Iran; 3 Behavioral Disorders and Substance Abuse Research Center, Institute of Mental Health and Addiction, Department of Nursing, Hamadan University of Medical Sciences, Hamadan, Iran; 4 Chronic Diseases (Homecare) Research Center, Department of Nursing, Hamadan University of Medical Sciences, Hamadan, Iran; 5 Health Sciences Research Center, Health Sciences & Technology Research Institute, Hamadan University of Medical Science, Hamadan, Iran; 6 Department of Midwifery, School of Nursing and Midwifery, Mother and Child Care Research Center, Hamadan University of Medical Sciences, Hamadan, Iran

**Keywords:** Domestic violence, Substance dependence, Suicide resilience, Child laborers

## Abstract

**Background:**

Child laborers are often defined as work that deprives children of their childhood, their potential and their dignity, and that is harmful to physical and mental development. Child laborers are one of the most vulnerable groups in domestic violence. Domestic violence severely affects the physical and mental health, and consequently affects substance dependence and resilience to suicide of these children. Accordingly, it is essential to examine domestic violence, substance dependence, and suicidal ideation in working children.

**Objectives:**

the present study aimed to investigate the relationship between exposure to domestic violence and substance dependence and suicide resilience on the other among child laborers in Iran.

**Methods:**

This study employed cross-sectional research. 600 child laborers were selected via convenience and snow ball sampling from one rehabilitation and welfare center and three charity organization societies in the west of Iran, from January to August 2022. They completed questionnaires. Data were analyzed by SPSS software version 22 and with using descriptive statistics (frequency, percentage, mean and standard deviation) and ANOVA, independent t-test and the multiple linear regression model with a backward strategy.

**Results:**

Findings showed that exposure to domestic violence has a strong and direct correlation with substance dependence (r = 0.94, p < 0.001) and strong and indirect correlation with suicide resilience (r =- 0.91, p < 0.001). Also substance dependence has a strong and direct correlation with suicide resilience (r = -0.87, p < 0.001) in child laborers. Variables of substance dependence, suicide resilience, gender, guardian’s disease status, living status and age can predict 76.51% of the variance in domestic violence in these children.

**Conclusion:**

Child laborers experience a lot of domestic violence, which severely affects their suicide resilience and substance dependence in them. Therefore, there is an urgent need for systematic support programs with content (teaching self-care behaviors, stress management, avoiding tense and violent environments) in order to support of these children and reduce domestic violence against them and subsequently reduce substance improve abuse resilience to suicide in these children.

## Background

Domestic violence means the violent and domineering behavior of a family member against another member or members of the same family [1]. A major social issue in many societies, domestic violence is defined as physical, psychological, or sexual violence against a family member and results in physical and/or psychological harms to that person [2, 3]. As the prevalence of domestic violence completely depends on cultures; yet, in most societies, vulnerable groups including the elderly, children and women are exposed to domestic violence more than others [2]. One of the most important groups susceptible to domestic violence is children [4]. In the United States, it is estimated that about three million families experience violence at least once a year, and an average of 3.3 million children are exposed in the family[5]. On the other hand, UNICEF reports that around 133–275 million children around the world witness domestic violence [6]. While some studies state that seeing scenes of violence and its consequences such as bruises on the body of family members and their depression and illness can be defined as exposure to violence [7, 8]. It is incontrovertible that considerable population of Iranian children is exposed to violence between their parents. As Vameghi et al. (8) asserted, 22.8% of the high school students in Tehran were exposed to physical violence between their parents[9]. Studies also state exposure to domestic violence is beyond the home environment, and any kind of violence in the surrounding environment, including school, and community is defined as domestic violence [8, 10].What is alarming is that parents are the main culprit in 70% of the cases of domestic violence against children [6, 7]. Younger children are more likely to be subject to domestic violence. These distressing statistics in Iran show that domestic violence must not be dismissed any longer and public awareness about it should be increased [7]. In Iran, any kind of physical, emotional and sexual violence against children is illegal in Iran, and every person in the society, especially teachers, employers, and families, are responsible for cases of violence and abuse of children (any kind of behavior by parents or others towards a child who harms his physical and mental health) to the police or social emergency center with the number 123[ 3,5].

However, there is no single causal factor related to domestic violence. Rather, scholars have concluded that there are numerous factors that contribute to domestic violence. Researchers have examined intersectional theory, attachment theory, exchange theory, identity theory, the cycle of violence, social learning theory, and victim-blaming theory in explaining domestic violence as theoretical perspectives [11–15]. Intersectional approach state Factors such as gender, race, wealth, etc. have caused some people to become powerful and others to become weak, and have expanded the field of inequality and injustice in the society [16]. Attachment theory helps to understand people’s behavior and explains how people’s experiences in childhood affect their interactions and behaviors in adulthood [13]. Another theory in the domestic violence literature is Lenore Walker’s cycle of violence. According to Walker, the cycle of violence is characterized by three distinct phases which are repeated over and over again in the abusive relationship. As a result, domestic abuse rarely involves a single isolated incident of violence. Rather, the abuse becomes a repetitive pattern in the relationship [14]. Exchange theory of family violence say that individuals will use force and violence in their relationships with intimates and family members if they believe that the rewards of force and violence outweigh the costs of such behavior [12]. Identity theory provides an important avenue for theoretical development in domestic violence research because all behavior, including aggression, is rooted in issues of self and identity [11, 15].

Obviously, the children who are forced to work are in more critical conditions, more prone to domestic violence, and more likely to display anti-social behaviors [17]. Although in Iran, it is forbidden to employ people under the age of 15, as well as to employ teenagers who are 15 to 18 years old, in hard jobs (working in factories, mines, bitumen spraying, sandblasting and welding or working with radioactive materials and radiation). But the sanctions have severely affected the economic conditions of the Iranian people. In the meantime, children in low-income families are forced to work as apprentices, waiters, salesmen in the shops of family members or others who experience violence, especially domestic violence, which severely affects their mental health [3]. According to Moayad et al. (2020) in Iran, a relatively high rate of violence and abuse of child in Iran occurs in work environments where child laborers is used and 77.6% of children have experienced at least one type of abuse. The most prevalent kind of abuse experienced by children is emotional abuse (70.4%), followed by neglect (52%), physical abuse (5.8%), and sexual abuse (3.6%) [18]. In addition, in Iran child exposure to domestic violence increase the likelihood of aggressive behaviors, anxiety, low self-confidence, delinquency, running away, suicide, and substance dependence in children and adolescents [19]. It is evident that child laborers are more prone to emotional issues and anti-social behaviors. Repeated acts of violence and physical, emotional, and even sexual abuse of children and adolescents who are subjected to forced labor incline this population toward substance dependence and suicide. The results of a study by Motazedian et al. (2020) in Iran showed that, respectively, 10.6%, 14.3%, and 1.8% of child laborers victims smoke, drink, and abuse drugs. In addition, domestic violence and substance dependence disturb adolescents’ emotional and psychological balance and increase suicidal tendencies in them [20]. Mhizha et al.(2020) in Zimbabwe found that substance dependence and emotional disorders following their infection with hepatitis and HIV are the main reasons for child laborers inclination toward suicide [21]. Other study express children exposed to domestic violence experience mental stress, depression and sadness and tend to use drugs for mental peace and to forget the violence [22, 23]. On a similar note, Toubaei et al. (2012) in Iran reported that feelings of guilt, depression, social isolation, and despair undermine the tolerance of adolescents who abuse drugs and create strong suicidal tendencies in them. This is despite the fact that child sex trafficking around the world are more exposed to violence and even domestic violence. Willis et al. (2018) states 1 million children are sex trafficked every year and the total number of prostituted children could be as high as 10 million. Prostituted children are at high risk of infectious disease, pregnancy, mental illness, substance dependence, and violence [24]. Thus, it appears that there is a close link between domestic violence and substance dependence and suicide resilience among adolescents, especially those who are subjected to forced labor [25].

Resilience is defined as a person’s ability to overcome problems using internal and external resources [26]. Resilience is an ability that people acquire during life and in dealing with problems. This is despite the fact that promoting resilience can be an important factor in preventing suicide in people in different societies [27, 28]. Resilience is defined as a person’s understanding of himself and resistance to difficulties [28]. In fact, an important factor for improving people’s tolerance and standing against difficult and exhausting conditions is resilience, which leads to a better understanding of the situation, more accurate analysis and finding more effective ways to solve problems, and increases people’s adaptability [29, 30]. Therefore, one of the most important factors related to suicide is resilience [31]. The concept of resilience against suicide has been proposed by Osman et al [32], the concept of suicide resilience refers to the ability and tolerance of a person in the face of terrible, life-threatening, painful, sudden and inappropriate events to deal with suicidal thoughts [10, 11]. Several qualitative and quantitative studies have addressed domestic violence and its impact on the physical and mental health of children and adolescents; yet, exposure to domestic violence of child laborers and how it correlates with substance dependence and suicide resilience among them has not been investigated. Accordingly, the two following aims were examined in study “evaluation the prevalence of child exposure to domestic violence, substance dependence and suicide resilience in child laborers” and “assessing the relationship between substance dependence and suicide resilience with child exposure to domestic violence in child laborers”. The hypotheses of this study included the following;” children exposed to domestic violence are more likely to use drugs”, “children exposed to more domestic violence have less resilience in the face of suicidal thoughts”.

## Methods

### Study design and setting

This study is a cross-sectional research. The conducted investigation is based on the strengthening the reporting of observational studies in epidemiology statement (STROBE), that is checklist for observational research.

### Participants and sampling

In this study sample size has been estimated base on study of Yaqubi doost et al. With β = 80% and α = 0.05 and taking into account the 20% drop about 700 samples [33]. Therefore, 700 child laborers were selected via convenience and snow ball sampling from one rehabilitation and welfare center and three charity organization societies in the west of Iran, from January to August 2022. The Welfare Organization is a government institution under the Ministry of Cooperation, Labor and Social Welfare that provides services to the needy people of the society and charity organization societies are established and managed by people and provide financial assistance, counseling, and treatment to low-income people for free. The information of protected persons is recorded in these centers. The researcher referred to these centers. The director of the child laborers center supported by the center introduced to the researcher those who were willing to participate in the study, then the researcher asked the children to invite their friends who are child laborers if they are willing to participate in the study. In order to increase the motivation to participate in the study, $2 was given to each person who completed and delivered the questionnaires. Based on the files of these children in these centers, these child laborers under the support of these centers were engaged in work such as working in shops, supermarkets, restaurants, car repair shops, peddlers, farms, wood-cutting workshops, pre-service in homes and construction workers. The inclusion criteria were: being willing to participate, literacy, Children 10–16 years, at least 12 months have passed since their working as child laborers and do not have a history of physical and mental disorders based on the doctor’s opinion and the medical file of these children. Child laborers who failed to answer more than half of the items on their questionnaires or did not return their questionnaires were excluded. The questionnaires are self-report, therefore, the participants were asked to complete the questionnaires include demographic characteristics, domestic violence against children, suicide resilience and Leeds dependence- should this be substance dependence. The majority of the questionnaires (72.12%) were completely gathered in April. 600 of the subjects completed questionnaires, Thus, the response rate was 85.71%. The participants’ reasons for not being completed in this study were lost the questionnaires and not motivated.

### Instrument

#### Demographic information questionnaire

The questionnaire included age, gender, number of family members, guardian’s disease status (physical or mental disease in the guardian of the children based on the file in the centers), living status and education.

#### Child exposure to domestic violence scale

Developed by Edleson et al. (2007), the Child Exposure to Domestic Violence Scale measures the extent of domestic violence against children. The scale consists of 33 items in five domains, namely involvement, level of violence, risk factors, community exposure, and victimization. The items are scored on a 5-point Likert scale (0–4). The maximum and minimum score limits are 99 and 0 respectively. Higher scores indicate the respondent’s greater exposure to domestic violence [10]. Rahimi et al. (2013) evaluated and verified the content, divergent, and convergent validity of the scale. The reliability of the scale was found to equal 0.86, which is a highly satisfactory value [34].

#### Leeds dependence questionnaire

Leeds Dependence Questionnaire was developed by Raistrick et al. (1994) in CIA to measure dependence on a wide variety of addictive drugs. The questionnaire consists of 10 items scored on a 4-point Likert scale (0–3). A score of under 10 indicates low dependence, 10 to 22 indicates moderate dependence, and above 22 indicates high dependence [35]. Messah et al. (2020) verified the content, divergent, and convergent validity of the questionnaire. The reliability of the scale was found to be a satisfactory 0.80 [36].

#### Suicide resilience inventory

Developed by Osman et al. in 2004, the suicide resilience inventory is a multi-dimensional suicide scale which evaluates the respondent’s perceived ability to cope with suicidal thoughts, access to external sources, and ability to deal with negative life events. The inventory consists of 25 items which address the dimensions of internal protection, external protection, and emotional stability. Scoring is based on a Likert scale: completely agree = 0, agree = 1, somewhat agree = 2, somewhat disagree = 3, disagree = 4, and completely disagree = 5. The score range is between 25 and 150, with higher scores indicating greater resilience against suicide [31]. The reliability of the entire questionnaire and its scales has been reported to range from 0.90 to 0.95. The validity of the instrument has been measured using different methods, all of which have shown it to be a valid scale [37].

### Data analyses

In this study, the collected data was analyzed with SPSS software version 22. For this purpose, descriptive statistics (frequency, percentage, mean and standard deviation) were used. ANOVA and independent t-test were also used to investigate the relationship between domestic violence with substance dependence and suicide resilience and demographic information in child labor. The significance level was considered P < 0.05. Then the demographic variables and suicide resilience and substance dependence with domestic violence (p < 0.25) were entered into the multiple linear regression model with a backward strategy. Regression model with a backward strategy is a stepwise regression approach that begins with a full (saturated) model and at each step gradually eliminates variables from the regression model to find a reduced model that best explains the data. The researcher evaluated before performing multiple linear regression, hypotheses including normality of data, homogeneity of variance, and independence of the residual.

### Ethics consecration

The study design was approved by the ethics committee of the Hamadan University of Medical Sciences (approval number: 1400.946). All experimental protocols were approved by Hamadan University of Medical Science and all methods were performed in accordance with the relevant guidelines and regulations. Also at the beginning of study the researcher introduced herself and explained the goals of the study and assured that all information would remain confidential and that they could withdraw from the study at any time. Finally, the written informed consent was obtained from all participants and from a parent and/or legal guardian, after providing them with sufficient information on the study. All methods were performed in accordance with the relevant guidelines and regulations.

## Results

### Demographic information

child laborers in this study were in the age ranging from 10 to 16 years with a mean age of 13.71 ± 2.16. Also, most of the children participating in this study was boys361 (60.16%), 254)42.34%) had a primary school’s degree and 203 (33.83%)of them were homeless. In Iran, access to cigarettes is much easier and cheaper than access to heroin, hashish, methamphetamine. However only 30 (5%) of the participants in this study did had not any drug abuse, but 71 (11.84%) of them only used cigarettes (about 10 cigarettes a day), Meanwhile, Meanwhile, 499 (83.16%) of them used heroin, salvia divinorum and opium in addition to cigarettes. About 201 (33.50%) children are addicted to heroin and 125 (20.84%) are addicted to opium and 173 (28.84%) are addicted to salvia divinorum. Also, child laborers as pre-service in homes and construction workers reported the highest exposure to domestic violence (71.12%). Findings also showed that there is a statistically significant relationship between domestic violence with being a gender and age, guardian’s disease status, living status (Table 1).

**Table 1:** The participants’ demographic characteristics and domestic violence score in child laborers

Demographic variables		N (%)	Domestic violence SD ± Mean	P Value
**Age (year)**	10–12	201(33.50)	93.12 ± 2.98	**0.038**
	12–14	274(45.66)	89.25 ± 3.47	
	14–16	125(20.84)	74.28 ± 3.61	
**Gender**	Boy	361(60.16)	81.43 ± 2.18	**0.018**
	Gail	239(39.84)	94.51 ± 3.47	
**Living status**	Homeless	203(33.83)	94.22 ± 2.58	**0.032**
	Single parent	134(22.34)	92.73 ± 3.14	
	Both parents	89(14.30)	76.34 ± 3.78	
	Other relatives	174(29.00)	89.74 ± 2.27	
**Education**	Literacy for reading and writing	124(20.66)	84.35 ± 3.04	0.478
	Primary school	214(42.34)	83.54 ± 2.74	
	Secondary school	173(28.84)	83.35 ± 2.51	
	High school	49(8.16)	82.97 ± 3.01	
**Guardian’s disease status**	No disease	101(16.83)	74.01 ± 2.72	**0.024**
	Physical	172(28.64)	87.46 ± 3.28	
	Mental	327(54.50)	92.38 ± 2.49	
**Number of family members**	2–4	39(6.50)	87.24 ± 3.41	0.37
	4–7	278(46.34)	88.47 ± 3.28	
	More than 7	283(47.16)	88.87 ± 3.12	

### Child exposure to domestic violence, substance dependence and suicide resilience in child laborers

All child laborers participating in this study reported exposure to domestic violence also 63% of them reported score of child exposure to domestic violence more than 72, which shows that more than half of the children in this study were exposed to more than average domestic violence. In addition, score of child exposure to domestic violence was 94.06 ± 3.45, substance dependence score was of 23.89 ± 2.14 and suicide resilience score was 98.36 ± 2.37 (Table 2).

**Table 2:** The means and standard deviations of the participants’ domestic violence, suicide resilience and substance dependence

Variable	dimensions	Mean ± SD (Each dimension)	Mean ± SD (Total)
**Domestic violence**	Level of violence	93.78 ± 3.15	**94.06 ± 3.45**
	Involvement	93.47 ± 3.78	
	Risk factors	94.58 ± 3.27	
	Community exposure	94.74 ± 3.32	
	Victimization	93.77 ± 3.74	
**Suicide resilience**	Internal protective	99.75 ± 2.32	**98.36 ± 2.37**
	External protective	97.27 ± 2.16	
	emotional stability	98.07 ± 2.64	
**Substance dependence**	Without dimension	23.89 ± 2.14	**23.89 ± 2.14**

### The relationship between child exposure to domestic violence, substance dependence and suicide resilience in child laborers

Findings in this study revealed that there is a strong and direct correlation between exposure to domestic violence and substance dependence (r = 0.94, p < 0.001) and strong and indirect correlation between exposure to domestic violence and suicide resilience (r =- 0.91, p < 0.001). Also, there is a strong and indirect correlation between substance dependence and suicide resilience (r = -0.87, p < 0.001) in child laborers (Table 3).

**Table 3:** Relationship between domestic violence, substance dependence, suicide resilience in child labor

**domestic violence**	**substance abuse**	**r = 0.94 p < 0.001**
**domestic violence**	**suicide resilience**	**r=-0.91 p < 0.001**
**substance abuse**	**suicide resilience**	**r=-0.87 p < 0.001**

### Predictors of child exposure to domestic violence in child laborers

The variables of substance dependence, suicide resilience, gender, guardian’s disease status, living status and age which had a p-value of smaller than 0.25 were entered into multiple linear regressions with the backward technique. These variables remained in the model and accounted for about 76.51% of the domestic violence variance in the child laborers (Table 4).

**Table 4:** The predictor variables domestic violence in child laborers

Factors		Non-standard coefficients		standard coefficients	T	Confidence intervals	P Value
		B	Standard error	β			
**Substance dependence**		3.18	2.11	0.50	1.50	0.41–1.74	0.001
**Suicide resilience**		2.76	1.09	0.65	2.53	0.37–1.86	0.001
**Age**		1.68	1.43	0.60	1.17	0.51–1.47	0.014
**Gender**	**Boy**	Reference	-	-	-		-
	**Gail**	1.42	1.24	0.35	1.14	0.27–1.14	0.026
**Guardian’s disease status**	**No disease**	Reference	-	-	-		-
	**Physical**	1.18	1.01	0.23	1.19	0.18–0.94	0.035
	**Mental**	1.14	1.03	0.22	1.10	0. 17-0.91	0.032
**Living status**	**Homeless**	Reference	-	-	-		-
	**Single parent**	1.08	1.02	0.21	1.05	0.17–0.87	0.030
	**Both parents**	1.10	1.04	0.23	1.05	0.18–0.74	0.033
	**Other relatives**	1.21	1.11	0.22	1.09	0.18–0.81	0.28
**Adjusted R2: 76.51%**							

## Discussion

Although the theories of domestic violence state that environmental conditions, health status, poverty, gender, age, colonialism and racism interact are factors that can affect the level of domestic violence [11–16], but so far any study has not investigated the relationship between exposure to domestic violence and substance dependence and suicide resilience in child laborers in Iran.

The findings of the present study showed that all child laborers in this study experienced exposure to domestic violence and 63% of them are exposure to high domestic violence and are highly prone to substance dependence while they have moderate resistance to suicide.

The findings of the study also showed that there is a significant, direct correlation between domestic violence and substance dependence and a significant, negative correlation between domestic violence and suicide resilience in child laborers victims. It was also found that substance dependence, suicide resilience, gender, guardian’s disease status, living status, and age predicted a high percentage of the domestic violence variance.

There were a number of studies on domestic violence, suicide resilience, and substance dependence in children and adolescents, but a review of literature showed that the rate of domestic violence, suicide resilience, and substance dependence in children subjected to child laborers had not been investigated. Accordingly, the authors used the findings of studies on domestic violence, suicide resilience, or substance dependence in children and adolescents to discuss the results of the present study.

In the present study, the domestic violence mean score of the child laborers victims was 94.06 ± 3.45, which demonstrated that the subjects had been exposed to high degrees of domestic violence. Also, there was a significant relationship between the child exposure to domestic violence with the variables of gender, age, living status and guardian’s disease status. Similarly, other research results show that child laborers victims experience considerable physical, sexual, and emotional violence in different cultures [4, 38, 39]. Seddighi et al. (2021) reported that, even though exposure to domestic violence in these children is high in the world, poverty, substance dependence, the health status of children and their guardians, exposure to child laborers, and children’s gender affect the extent of violence against children, which is consistent with the findings of the present study. In Iran, child laborers girls were more exposer to violence [40]. On a similar note, Gibbs et al. (2021) found that violence against children, child abuse, child laborers trafficking, and sexual abuse of children are high and supportive and preventive measures should be taken to reduce violence against children and improve their physical, sexual, and mental health [41]. In their study, Carneiro et al. (2017) concluded that domestic violence impairs children’s academic performance, undermines their self-confidence, and encourages them to abuse drugs [42]. Moreover, Catani et al. (2008) stated that the rate of violence against children in Afghanistan and Sri Lanka is very high. The children’s guardians force them to perform physically demanding tasks which cause many physical injuries to the children. Also, the children’s guardians in these countries often use the children to trade and smuggle drugs, which sometimes results in the children’s being arrested, convicted, and imprisoned. Female children are more likely to be exposed to physical and sexual violence and have their rights violated. Accordingly, policy-makers should pass stricter laws to defend children’s rights [38]. Obviously, as a result of the difficult conditions in their lives, child laborers victims are more prone to violence, and are, therefore, in more urgent need of comprehensive support.

The substance dependence mean score of the child laborers victims in the present study was a high 23.89 ± 2.14. It was also found that there is a significant and direct correlation between substance dependence and domestic violence and a significant and negative correlation between substance dependence and suicide resilience in children subjected to child laborers. On a similar note, Khairi et al. (2016) found that anti-social behaviors, aggression, stress, and substance dependence are highly high among child laborers victims, and the children who have been exposed to domestic violence, especially sexual violence and abuse, are more aggressive and inclined to abuse drugs [43]. Thus, it appears that there is a direct correlation between violence, anti-social behaviors, aggression, and substance dependence. Similarly, Moayad et al. (2021) reported that the rate of physical, psychological, and sexual violence against child laborers victims is high, and there is a direct relationship between violence, especially sexual and psychological violence against this population and the incidence of anti-social behaviors and substance dependence among them [18]. Feeny et al. (2021) stated that, even though physical violence against child labor victims has been somewhat investigated, psychological violence and its impact on the mental health of this population has not been subject to much research. Psychological violence has a significant role in the emergence of anti-social behaviors, aggression, depression, and inclination to substance dependence among children who are forced to work. Thus, more extensive investigation and support is needed to improve the health of child labor victims [44].

The suicide resilience mean score of the subjects of the present study was found to be 98.36 ± 2.37, which is considered moderate. In addition, there was a negative correlation between suicide resilience on the one hand and domestic violence and substance dependence on the other. However, Susan et al. (2020) found that adolescents and young adults in Bhutan have very low suicide resilience. In this population, poor adolescents who abuse drugs and are exposed to violence and stress are more suicide prone [45]. Even though, as with the present study, the above-mentioned study reported that adolescents have low suicide resilience, the present study evaluated resilience in children and adolescents exposed to child labor. It is evident that this population has lower suicide resilience than adolescents and young adults who live in more comfortable family environments.

As with the present study, the study of Gallagher et al. (2018) showed that children and adolescents’ suicide resilience is a direct function of their living conditions, the quality of their interactions with their parents, their financial status, and peer groups. Children who live in unsatisfactory conditions, do not have proper commutation with their parents, and are exposed to violence by their parents have less suicide resilience [46]. Likewise, Kapoor et al. (2018) reported that exposure to violence and physical and sexual abuse during childhood has very adverse effects on the mental health of individuals in adolescence and adulthood and substantially increases suicidal tendencies in the victims [47]. Even though these findings are consistent with the results of the present study, Kapoor et al. evaluated suicidal tendencies in adolescents and adults who had been subjected to domestic violence in childhood, while the present study addressed child labor victims, who often live in difficult conditions and are more prone to violence and anti-social behaviors.

It was also found that the variables of substance dependence, suicide resilience, gender, guardian’s disease status, living status, and age predicted 76.51% of the variance of domestic violence against child labor victims. On a similar note, a study by Martins et al. (2019) showed that substance dependence, poverty, having a single parent, and a poor academic status are the most important predictors of domestic violence among children and adolescents [48]. Despite the similarity between the predicting variables identified by the two studies, the present study investigated domestic violence against children exposed to child labor, and, thus, the predicting variables of domestic violence included suicide resilience, the gender and age of the children, and the guardians’ disease status in addition to the variables reported by Martins et al. On the other hand, Cuartas et al. (2019) found that poverty, residence, and financial status are the most important predictors of domestic physical violence against children. The researchers suggested that supportive measures should be taken to reduce physical violence against children, which will in turn improve the health of children, especially those who live in adverse conditions, e.g. child labor victims [49]. According to Rubenstein et al. (2017), the variables of drug and alcohol abuse, income/financial status, mental health/coping strategies, and limited social support are the main predictors of violence against children, which agrees with the findings of the present study [50]. The similarities between the present study and the above-mentioned studies can be attributed to the fact that they all address domestic violence against children and the factors which contribute to this type of violence are relatively the same in most societies. However, the higher estimates of domestic violence in the present study as compared to the other studies can be accounted for by the fact that this study investigated the status of children who are subjected to child labor, who are exposed to more violence as a result of their living conditions.

Finally, the findings of the present study showed that child labor victims suffer from domestic violence and substance dependence and have moderate suicide resilience, that effect on their physical and mental health. Accordingly, there is an urgent need for systematic support programs with content (teaching self-care behaviors, stress management, avoiding tense and violent environments) regularly in the long term can effect to reduce exposure to domestic violence in these children.

### Limitations

One of the major limitations of the study was the children’s failure to return the questionnaires due to lack of motivation or losing the questionnaires. Therefore, it is suggested that future research address exposure to domestic violence, substance dependence, and suicide resilience among child laborers in different societies and larger samples to acquire a more accurate understanding of exposure to domestic violence in child labor. Another limitation of this study is the collection of information using only a questionnaire, so it is suggested to use other methods of information collection, including interviews and observations, in future studies. One of the other limitations was mediator and moderator variables, because there are many mediator and moderator variables in the assessment of domestic violence in child labor, and the present study only examined the effects of some of them. Therefore, it is suggested to investigate the effect of other mediator and moderator variables in future studies. One of the limitations of the current study was not examining the cause of domestic violence and the cause of substance dependence in child laborers. Based on this, it is suggested that future studies investigate the cause of domestic violence and the cause of substance dependence in working children.

## Conclusion

Children who are subjected to child labor experience high degrees of domestic violence, with extremely adverse effects on their physical and mental health, resilience, and resistance to substance dependence. Substance dependence, suicide resilience, gender, age, guardian’s disease status and living status are influential factors in the rate of domestic violence against this population: they predicted 76.51% of the variance of domestic violence against child labor victims. Also, Child laborers as housemaids and construction workers reported the highest exposure to domestic violence (71.12%). Based on this, it is necessary for the welfare officials and public health audience to prevent the employment of children as workers, especially in these environments, and reduce the exposure of children to domestic violence by careful monitoring and frequent visits.

## Data Availability

The datasets used and/or analyzed during the current study are available from the corresponding author on reasonable request.
