# Correction to: Child exposure to domestic violence, substance dependence and suicide resilience in child laborers

**DOI:** 10.1186/s12889-023-16175-9

**Published:** 2023-10-02

**Authors:** Fateme Mohammadi, Khodayar Oshvandi, Farshid Shamsaei, Masoud Khodaveisi, Salman Khazaei, Seyedeh Zahra Masoumi

**Affiliations:** 1 Chronic Diseases (Home Care) Research Center and Autism Spectrum Disorders Research Center, Department of Nursing, Hamadan University of Medical Sciences, Hamadan, Iran; 2 Mother and Child Care Research Center, Department of Nursing, Hamadan University of Medical Sciences, Hamadan, Iran; 3 Behavioral Disorders and Substance Abuse Research Center, Institute of Mental Health and Addiction, Department of Nursing, Hamadan University of Medical Sciences, Hamadan, Iran; 4 Chronic Diseases (Homecare) Research Center, Department of Nursing, Hamadan University of Medical Sciences, Hamadan, Iran; 5 Health Sciences Research Center, Health Sciences & Technology Research Institute, Hamadan University of Medical Science, Hamadan, Iran; 6 Department of Midwifery, School of Nursing and Midwifery, Mother and Child Care Research Center, Hamadan University of Medical Sciences, Hamadan, Iran

**Correction to: BMC Public Health (2023) 23:467**

10.1186/s12889-023-15367-7

The original publication of this article contained two instances of an incorrect ethic code. The incorrect and correct information is listed in this correction article. The original article has been updated.

Incorrect approval number: 1400.524

Correct approval number: 1400.946

